# Non‐Operative Management of a Complete ACL Rupture With the Cross‐Bracing Protocol in an Elite Australian Rules Football Athlete

**DOI:** 10.1002/ccr3.72818

**Published:** 2026-05-31

**Authors:** Keiley Mead, Matthew Dowsett, Zoe Cass, Stephanie Filbay

**Affiliations:** ^1^ Discipline of Medical Imaging Science, Faculty of Medicine and Health The University of Sydney Sydney Australia; ^2^ Department of Orthopaedic and Trauma Surgery Addenbrooke's Hospital, University of Cambridge Cambridge UK; ^3^ The Stadium Sports Medicine Clinic Sydney Australia; ^4^ Centre for Health, Exercise and Sports Medicine, Department of Physiotherapy University of Melbourne Sydney Australia

**Keywords:** anterior cruciate ligament injuries, conservative treatment, knee injuries, knee joint, magnetic resonance imaging

## Abstract

Anterior cruciate ligament (ACL) injuries are traditionally managed with surgical reconstruction, but the ACL's healing capacity continues to be explored. Non‐operative management strategies have emerged as viable alternatives for ACL‐injured patients either via rehabilitation alone or approaches like the Cross‐Bracing Protocol (CBP). We report the case of a female Australian rules football (AFL) athlete with a complete ACL rupture sustained through a non‐contact hyperextension mechanism. The athlete was treated with the 12‐week CBP, and the serial MRI examinations at 3‐, 6‐, 12‐, and 36‐month post‐injury are presented, demonstrating progression from a complete ACL rupture to a contiguous ACL with normal thickness, volume, trajectory, and signal intensity. Patient‐reported outcome measures (PROMS) were collected at 3‐, 6‐, 12‐, and 36‐month post‐injury. The athlete successfully returned to elite‐level AFL sport one year post‐injury and reported no re‐rupture at 3 years post‐injury. This case demonstrates the potential utility of the CBP to restore normal ACL anatomy and result in stability of the knee following non‐surgical management.

## Graphical abstract

Longitudinal MRI assessment of a complete ACL rupture in a 20‐year‐old female athlete managed non‐operatively with the Cross Bracing Protocol: Proton density fat saturated (PD‐FS) sequence MRI of the left knee in the sagittal plane with complete ACL rupture at day 3 post injury. (A) showing ACL fibre discontinuity (blue arrow) and pivot shift pattern of bone marrow oedema signal (red arrow). Follow up imaging was completed at 3‐months (B) showing reduced thickness at the femoral ACL attachment (yellow arrow) with a sag in the ACL (orange arrow), at 6‐months (C) showing minimal reduction in the thickness of the ACL at the femoral attachment (teal arrow), at 12‐months (D) showing a taut ACL with low signal intensity along the length of the ACL (pink arrow), and at 36‐months (E) showing an intact, taut ACL with normal low signal intensity (purple arrow).

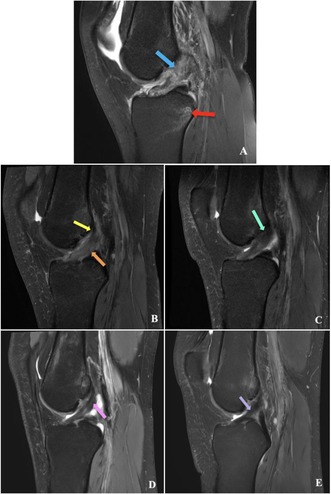

**Key Clinical Messages (KCM)**

Following a complete ACL rupture, non‐operative anterior cruciate ligament (ACL) injury treatment with the Cross‐Bracing Protocol (CBP) resulted in successful return to play in an elite athlete participating in a high‐impact contact sport, with restoration of ACL continuity on MRI and patient‐reported functional stability.

## 1 Introduction

The prevalence of anterior cruciate ligament (ACL) injuries continues to rise, especially in Australia. More than 91 per 100,000 people under the age of 25 suffer an ACL injury, and this number is believed to be under‐reported [1, 2]. It was previously thought that the ACL was unable to heal, and as such, ACL reconstruction (ACLR) has remained the most common management for ACL injury [3]. Australia has one of the highest population‐based incidences of ACLR surgery, and from 2001 to 2020, there was a significant increase in adult ACLR in Australia [4, 5]. In the elite athlete population, it is considered mainstream to opt for surgical intervention following an ACL injury, with many individuals having limited awareness of trialing rehabilitation alone or other viable non‐surgical alternatives [6].

The Cross‐Bracing Protocol (CBP) is a novel non‐operative treatment approach for acute ACL injuries. The protocol challenges the long‐held belief that ruptured ACLs cannot heal and offers an alternative to early surgical reconstruction in selected patients [7]. Evidence continues to grow in support of the ACL's healing capacity, either spontaneously or facilitated by non‐operative bracing protocols [7, 8, 9]. Only one original research paper has been published to date that assesses outcomes of the CBP for non‐operative management of ACL injuries, which found that 90% of participants had evidence of restored ACL continuity, considered a sign of ACL healing, on their 3‐month follow‐up MRI [9]. This result suggests promising outcomes with this non‐operative method for supporting restoration of ligament continuity and structure, warranting further investigation to determine the appropriateness of this treatment method for use in elite athletes.

Current evidence suggests that for ACL‐injured patients, the exploration of non‐operative interventions prior to surgical intervention for individuals is a practice that should be adopted by clinicians, only shifting to surgical interventions if acceptable function cannot be achieved [10, 11]. Despite this evidence, no intervention or delayed surgical or non‐surgical interventions may result in ACL deficiency, potentially increasing the risk of secondary injury such as meniscal damage due to recurrent instability episodes [12]. Over the last few years, there have been further improvements in the scientific community's awareness of non‐operative pathways for ACL‐injured patients, yet their application in elite athlete populations remains underreported and lacks long‐term data. Regardless of surgical or non‐surgical interventions, ACL injuries take an extreme toll on the physical, mental, and emotional well‐being of elite athletes [13]. The uncertainty regarding the appropriateness of non‐operative treatments for ACL injuries in elite athletes can be attributed to the scarcity of studies investigating management options such as the CBP in this population. Studies are difficult to conduct in elite athletes due to external considerations that are specific to high‐level athletes, such as performance pressure, financial considerations, and contracts, which can affect athletes' decision‐making in healthcare [14, 15]. For this reason, case studies in elite athletes with ACL injuries managed non‐surgically can be useful in understanding the potential of these management options in this population [16, 17, 18]. This case report describes the experience of an elite Australian athlete who underwent the CBP for management of an acute ACL rupture and can be utilized to generate conversation between the athlete and healthcare providers about the potential of non‐operative treatments for ACL injuries in elite athletes.

## 2 Case History/Examination

### 2.1 Presenting Complaints and Medical History

A 20‐year‐old female Australian rules football (AFL) athlete with no significant medical history presented to the emergency department following a non‐contact hyperextension knee injury after stepping into a pothole during AFL competition. Immediately following the incident, the athlete reported significant pain, an inability to weight bear, a sensation of knee instability, and reported hearing an audible ‘pop’. Supine non‐weight‐bearing anterior‐to‐posterior (AP) and lateral X‐rays of the left knee were performed, which showed no evidence of bony injury. The on‐field physiotherapist confirmed a positive Lachman test and a positive pivot shift test at the time of injury. On examination, the emergency department physician identified a potential soft tissue knee injury. The athlete was subsequently discharged with the following management plan:

- Ice application to the knee for a maximum of 20 min at a time
- Rest and use of crutches to weight‐bear as tolerated
- MRI referral if symptoms persisted after resolution of swelling
- Follow up with a general practitioner (GP) or physiotherapist

### 2.2 Initial Imaging & Diagnosis

**FIGURE 1:**
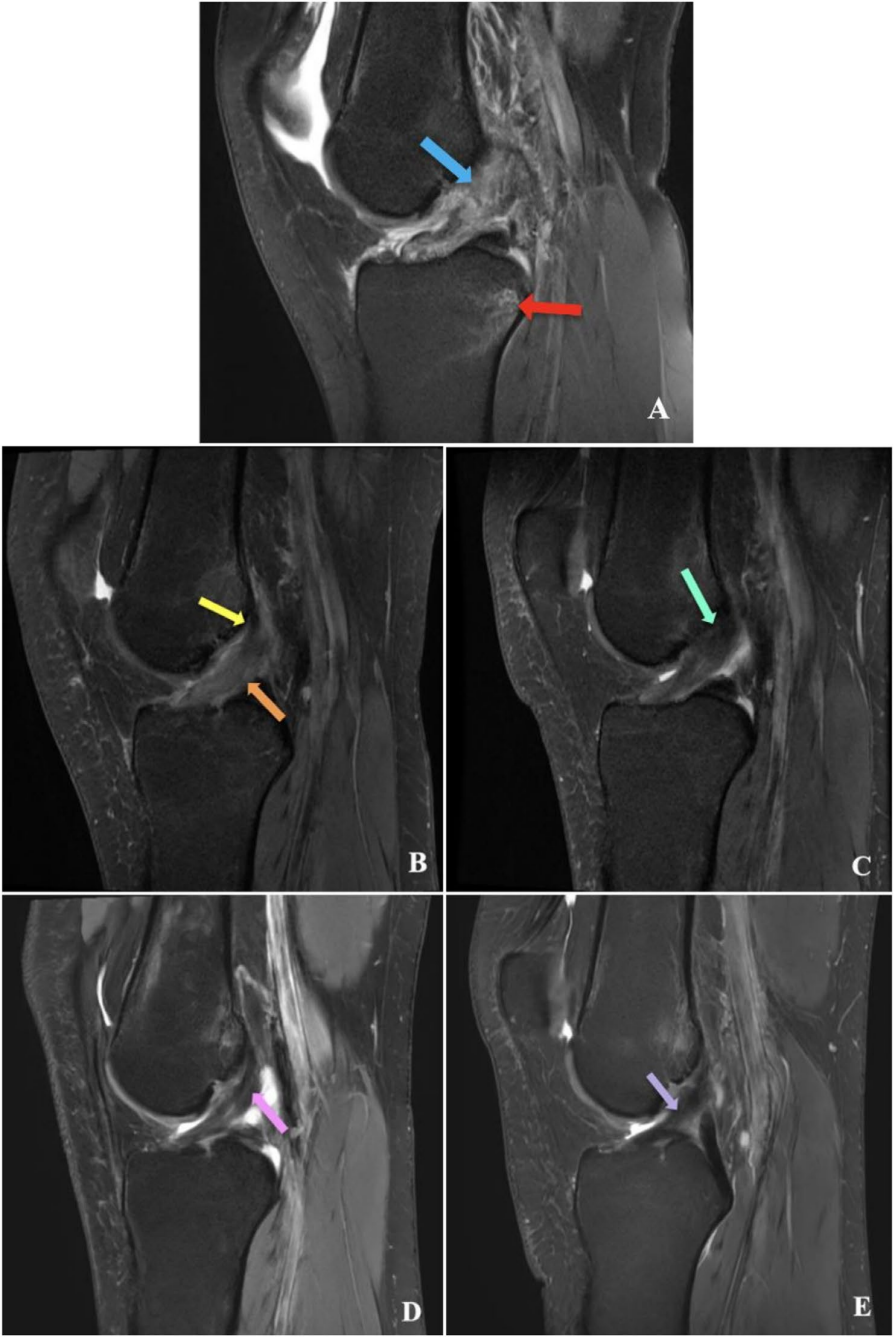
Proton density fat saturated (PD‐FS) sequence MRI of the left knee in the sagittal plane of a 20‐year‐old female athlete with complete ACL rupture at day 3 post injury. (A) showing ACL fiber discontinuity (blue arrow) and pivot shift pattern of bone marrow oedema signal (red arrow). Follow up imaging was completed at 3 months (B), showing reduced thickness at the femoral ACL attachment (yellow arrow) with a sag in the ACL (orange arrow); at 6 months (C), showing minimal reduction in the thickness of the ACL at the femoral attachment (teal arrow); at 12 months (D), showing a taut ACL with low signal intensity along the length of the ACL (pink arrow); and at 36 months (E), showing an intact, taut ACL with normal low signal intensity (purple arrow).

Three days post‐injury, the athlete underwent an MRI scan at a local private clinic. The athlete's MRI was reviewed by a senior musculoskeletal radiologist who reported an acute full‐thickness rupture of the ACL, a pivot shift pattern of bone marrow oedema signal, and a small hemarthrosis with pericapsular oedema signal. There was partial tissue displacement of the distal remnant of the ACL, which was located under the lateral femoral condyle as seen in Figure 1A.

### 2.3 Sports & Exercise Medicine Physician Consultation

On day eight post‐injury, the athlete presented for a clinical examination with a sport and exercise medicine (SEM) physician. The Lachman test and pivot shift test were not repeated; however, significant effusion was clinically confirmed. Operative interventions, including ACLR, and non‐operative interventions, including rehabilitation alone and the CBP, were discussed with the athlete in detail. The athlete had access to the Australian Medicare system as well as private health insurance coverage and, therefore, could access early ACLR surgery. The athlete was provided with information regarding the CBP and sent home to discuss all ACL management options with their family.

## 3 Methods (Differential Diagnosis, Investigations, and Treatment)

On day nine post‐injury, with a clear understanding of the risks and benefits of the different treatment options presented, the athlete expressed their desire to attempt an alternative non‐operative management approach in an attempt to facilitate ACL healing, the CBP. The athlete gave informed consent to commence the CBP using a range‐of‐motion (ROM) brace for a 12‐week period under the guidance of an accredited physiotherapist. The patient's management strategy, including the 12‐Week Cross‐Bracing Protocol and specific rehabilitation exercises, is summarized in Table 1. An ultrasound lower limb venous doppler was performed, which revealed no evidence of deep vein thrombosis (DVT). The athlete was fitted with a ROM brace and opted to have a second ROM brace for use when swimming. Once the athlete was fitted with the ROM brace in 90 degrees of knee flexion, they were commenced on a course of one 10 mg tablet of Rivaroxaban per day, an anticoagulant, to mitigate potential risks of DVT during the first 8 weeks of the CBP. The athlete opted to utilize elbow crutches to mobilize during the CBP until weight‐bearing was able to be safely conducted, and the patient had access to a wheelchair, which they decided to also utilize in the first 6 weeks of the CBP when traveling large distances, for example, on long walks with friends, or to the grocery store to shop with family. The patient utilized a plastic chair on a non‐slip mat with the brace removed to shower during the protocol.

**TABLE 1:** Overview of the patient's management strategy, including the 12‐week Cross‐Bracing Protocol (CBP).

Week of cross‐bracing protocol	Brace range of motion (ROM)	Overview of rehabilitation exercises
1–4	ROM brace locked at 90°	– Quadriceps and hamstring co‐contractions – Calf TheraBand plantarflexion and seated calf raises – Hip abduction and extension – Contralateral limb; single leg press, leg extension, hamstring curls, calf raise, glute bridge, core activation – Foot/ankle “calf pump” exercises to mitigate the risk of DVT
5	ROM brace locked at 60°–90°	– Quadriceps and hamstring co‐contractions at varying angles – Continue Calf TheraBand plantarflexion and seated calf raises – Hip abduction and extension with ankle weights or TheraBand – Hamstring and gluteal bridges – Continue contralateral limb strength; single leg press, leg extension, hamstring curls, calf raise, glute bridge, core activation – Continue Foot/ankle calf pump exercises to mitigate the risk of DVT
6	ROM brace locked at 40°–90°	
7	ROM brace locked at 30°—full flexion	– As above plus; – Wall squats/holds (Week 7 = 30°, Week 8 = 20°) – Leg press double leg +/− single leg (available range)‐Body weight squats within brace limits – Begin stationary bike‐Knee range of motion exercises
8	ROM brace locked at 20°—full flexion	
9	ROM brace locked at 10°—full flexion	– Knee range of motion exercises‐Leg press (single leg) – Body weight squats – Bridges‐ hamstring and gluteal – Crab walks/monster walks – Calf raises – Static balance exercises
10–12	Unrestricted ROM brace	– As above plus; – Hamstring curl machine.
13–14	No ROM brace. No other knee brace was utilized post‐CBP	– Continue hamstring and quad strength – Addition of single‐leg squatting/Bulgarian squats – Lunges (half range) – Dynamic balance exercises – Jogging on the spot – Light skipping
15–16		– Continue previous strength and dynamic balance exercises – Addition of walking lateral movements, e.g., crab walking, side stepping, grapevines, etc. – Addition of jumping/landing exercises – Group exercises classes including plyometrics such as single leg drops, countermovement jumps, step up jumps, single leg lands, etc. – Running (straight line only)
17		– Faster straight line running – Single‐leg hopping drills – Continue jumping/landing drills – Continue plyometric and strength exercises for the lower body
18		– Agility – Introduce multidirectional hopping/landing drills in plyometric classes – Continued plyometric and strength exercises for the lower body
26		– Return to run program commenced in a group class format on a grass field twice per week. The athlete progressed by increasing the time of runs and the intensity of runs each week – Continued plyometric and strength exercises for the lower body
38		Returned to non‐contact fitness training with the AFL team for pre‐season training
52		Passed return to sport testing and returned to full‐contact AFL training
61		Returned to the AFL pre‐season match

*Note:* AFL = Australian Rules Football; DVT = Deep vein thrombosis; ROM = range of motion.

After 8 weeks, the athlete was mobilizing without crutches and no longer required anti‐coagulation. Throughout the rehabilitation period, the athlete followed a comprehensive diet plan that prioritized anti‐inflammatory foods and a high‐protein intake in consultation with an accredited sports dietician. Due to gluten intolerance, the athlete maintained a strict gluten‐free diet. It was normal for this athlete to abstain from alcohol, smoking, vaping, and other illicit substances; however, these behaviors were suggested by the team to encourage optimal biological healing outcomes. In consultation with a qualified sports dietitian from weeks 12–52 of the CBP, the athlete supplemented their rehabilitation regimen with hydrolyzed collagen, taking three tablets per day containing 1,000 mg hydrolyzed collagen and 100 mg ascorbic acid (Vitamin C) before exercise rehabilitation sessions.

A milestone‐based approach was adopted for guiding the progression of rehabilitation and exercise, which was overseen by a team consisting of an experienced sports and exercise physician, a physiotherapist, and professional sports trainers. From day 0 to 3 months after commencing the CBP, the athlete participated in daily physical training under the supervision of a physiotherapist or exercise physiologist using the same goal‐based progression criteria as post‐ACLR patients at the clinic. From week 20 onwards, the athlete was occasionally supervised by professional sports trainers at their AFL club who worked closely with the athlete's physiotherapist and sports & exercise physician as they returned to training. Throughout the athlete's rehabilitation period, the patient underwent clinical testing, including range of motion (ROM) measurements, a Lachman test, effusion assessment, and assessment of the patellofemoral joint (PFJ), as outlined in Table 2. Additionally, patient‐reported outcome measures (PROMS), including the International Knee Documentation Committee Subjective Knee Form (IKDC) and the ACL Return to Sport after Injury scale (ACL‐RSI), were collected and are presented in Table 2. The athlete was assessed with functional assessments presented in Table 3, which were considered when determining the appropriateness of the athlete's return to sport.

**TABLE 2:** Clinical testing and patient reported outcome measures (PROMS) over a three‐year period following ACL injury.

Time point		3 month testing		6 month testing		12 month testing		36 month testing	
Side Measured		Injured Side	Uninjured Side	Injured Side	Injured Side	Injured Side	Uninjured Side	Injured Side	Uninjured Side
Physical Examination	Knee Extension AROM	−4^o^	10^o^	1^o^	5^o^	2^o^	5^o^	2^o^	–4^o^
	Knee Flexion AROM	119^o^	149^o^	141^o^	160^o^	142^o^	147^o^	143^o^	149^o^
	Lachman Test^a^	Grade 1	Grade 0	Grade 0	Grade 0	Grade 0	Grade 0	Grade 0	Grade 0
	Knee Effusion	Mild	Nil	Nil	Nil	Nil	Nil.	Nil.	Nil.
Patient Reported Outcome Measures	IKDC^b^	50/100		91/100		100/100		100/100	
	ACL‐RSI^c^	6.7/100		79.5/100		100/100		100/100	

Abbreviations: ACL‐RSI = The Anterior Cruciate Ligament—Return to Sport after Injury scale; AROM = active range of motion; IKDC = International Knee Documentation Committee Subjective Knee Form.

^a^ The Lachman test is a clinical examination performed to evaluate the integrity of the anterior cruciate ligament (ACL). The knee is placed in slight flexion with a manual anterior force applied to the proximal tibia to assess anterior tibial translation and endpoint quality, graded as either 0 (denoting a firm endpoint and < 3 mm anterior translation), 1 (denoting mild laxity with 3–5 mm anterior translation and a firm endpoint), 2 (indicating moderate laxity with 5–10 mm of anterior translation and a soft endpoint), or 3 (severe laxity with > 10 mm of anterior translation and an absent or very soft endpoint). The patient's Lachman test results demonstrate a reduction in anterior tibial translation over the period from acute injury to the 3‐month assessment, which remained stable across the athlete's rehabilitation and return to sport.

^b^ International Knee Documentation Committee (IKDC) is a scoring system that evaluates knee symptoms, function, and sports activity to assess outcomes after knee, or ACL, injury or treatment. A higher score indicates better knee function and fewer limiting symptoms. The patient's IKDC scores over the follow‐up period indicate progressive improvement in subjective knee function and symptom relief, stabilizing at the 12‐month assessment when the athlete returned to sport and remained unchanged at the 36‐month assessment. These results reflect the patient's perceived knee stability, symptoms, and ability to perform their regular activity without limitation.

^c^ The ACL‐RSI is a patient‐reported outcome measure that evaluates psychological readiness to return to sport after ACL injury or reconstruction, with a higher score indicating greater psychological readiness for returning to regular activity. The patient's ACL‐RSI scores over time reflect progressive improvement in psychological readiness and confidence to return to sport, peaking at the 12‐month assessment and remaining stable at 36 months post‐injury, similar to the IKDC.

**TABLE 3:** Attainment of return to sport functional assessment goals.

Functional assessment	Goal for passing return to sport testing	3 months post ACL injury	6 months post ACL injury	9 months post ACL injury	12 months post ACL injury
Y Balance test	< 4 cm and > 90% LSI^a^				
Dynamometer measured Isometric strength test—knee Extension at 30°	> 90% LSI				
Dynamometer measured Isometric strength test—knee Flexion at 30°	> 90% LSI				
Quadriceps/Hamstring Strength Ratio	> 80% LSI				
Dynamometer measured Isometric strength test—knee Flexion at 90°	> 70% LSI				
Hip abduction Dynamometry (Supine)	> 90% LSI				
Single leg Decline Squats	> 90% LSI				
Single leg Hamstring Bridge	> 90% LSI				
40 cm lateral Hops (30 s)	> 40 repetitions				
Hop for distance	> 90% LSI				
Triple hop for distance	> 90% LSI				
Double leg CMJ (Force Plates)	Height: > 25 cm Contact Time: < 0.2 s RSI^b^: > 1.25 m/s				
Single leg CMJ (Force Plates)	Height: > 90% LSI Asymmetry: < 10%				
Drop vertical jump 30 cm (Force Plates)	Height: > 30 cm Contact Time: < 0.2 s RSI: > 1.25 m/s				
Drop vertical hop 20 cm (Force Plates)	Height: > 15 cm Contact Time: < 0.28 s RSI: > 1.55 m/s				
Squat 3–5 RM	> 1.5× BW				
Deadlift 3–5 RM	> 1.5× BW				
Single leg‐leg press	> 1× BW				
Split Squat 3–5 RM	> 1.5× BW				
Planned and unplanned 45°CoD	Pain free				
Planned and unplanned 90°CoD	Pain free				

*Note:* Green shading indicates that the patient achieved the specific target goal for passing return to sport (RTS) criteria at that respective testing checkpoint.

Abbreviations: ACL = anterior cruciate ligament; BW = body weight; CMJ = counter movement jump; CoD = change of direction; LSI = limb symmetry index; RM = repetition maximum; RSI = reactive strength index.

^a^ Limb Symmetry Index (LSI) is the ratio of performance of the injured limb to the uninjured limb, expressed as a percentage, used to assess recovery in strength and functional testing after ACL injury. An LSI of > 90% is the target, as this number suggests minimal limb asymmetry. LSI results of < 90% often indicate residual deficits in the strength of function of the injured knee. The patient's ability to meet the target LSI over time is suggestive of progressive improvement toward symmetrical limb performance, achieving three LSI targets at the six‐month assessment, followed by all LSI targets by the nine‐month assessment.

^b^ The Reactive Strength Index (RSI) is a performance measure calculated as jump height in meters (m) divided by ground contact time in seconds (sec) during a plyometric test, used to assess lower‐limb explosive strength and stretch–shortening cycle efficiency. A low RSI suggests deficits in neuromuscular performance or reactive strength. The patient's RSI trend over time indicates progressive improvements in plyometric performance, achieving all RSI targets at the 9‐month assessment except for the drop vertical jump from 30 cm measured with force plates, which was achieved at the 12‐month assessment prior to the athlete returning to sport.

## 4 Results

The athlete was consulted by a sports and exercise physician via telehealth every two weeks during the 12‐week CBP with no reported complications. The athlete was compliant with all medication, diet, and exercise recommendations and was regularly assessed by their physiotherapist with universally accepted criteria‐based staged ACL rehabilitation.

### 4.1 3‐Month Follow‐Up

At 3 months after commencing the CBP, the athlete underwent a follow‐up MRI with the results suggesting that the athlete had a healing ACL which was contiguous from its femoral and tibial footprint (see Figure 1B). However, there was a slight deviation in the ACL's trajectory, relative to Blumensaat's line. There was a reduced thickness and volume at the femoral origin of approximately 20%, consistent with the acute pattern of ACL injury described (i.e., partial avulsion of the ACL tissue at the femoral origin). There was a minor low signal intensity focus lateral to the tibial footprint of the ACL, which could represent a small remaining segment of displaced ACL tissue. The pivot shift pattern of bone marrow oedema signal visible on the acute MRI had resolved. No other knee injuries were present on MRI. At the time of brace removal, the athlete had a slight extension active ROM deficit of approximately 10°, no effusion, a negative Lachman test, and a negative pivot shift test.

### 4.2 6‐Month Follow‐Up

At 6 months post injury, the athlete underwent a follow‐up MRI with similar morphological findings to the 3‐month MRI (see Figure 1C). Whilst collagen maturation of the ACL cannot be confirmed without histopathologic analysis, it was hypothesized that the ACL had undergone collagen remodeling and maturation as the MRI showed increased homogeneity of the MRI signal intensity along the course of the ACL. Clinically, the athlete had a negative Lachman test. In contrast to the 3‐month assessment, at 6 months there was a very slight glide when performing the pivot shift test on the left knee, which was attributed to the subtle deviation of the ACL from Blumensaat's line, hypothesized to represent a slight elongation of the ACL where the rupture occurred. The athlete lacked approximately 5° of terminal knee extension and similarly 5° of end knee flexion. The athlete self‐reported a ‘click’ in the knee upon extension. The musculoskeletal radiologist on site was consulted and believed that the displaced tissue under the lateral femoral condyle had been largely reabsorbed; however, a very small piece of tissue was observed on a 6‐month MRI that may have acted as a small cyclops lesion, potentially explaining the ‘clicking’ sensation. The athlete's rehabilitation was continued.

### 4.3 12‐Month Follow‐Up

A 12‐month follow‐up, the MRI demonstrated contiguous ACL healing with the same morphological features as observed at 6 months, but with no elongation and normalization of the signal intensity throughout the entire course of the ACL (see Figure 1D). Clinically, there was no side‐to‐side difference in passive knee laxity with another negative Lachman test and a negative pivot shift test. The athlete reported that the ‘click,’ noted at the last check‐up, had resolved. Given the above MRI findings, the athlete was deemed to have achieved “biological readiness,” and given they had passed “return to sport” functional testing with a physiotherapist, the athlete thereafter returned to AFL at their previous level.

### 4.4 3‐Year Follow‐Up

At 3 years post ACL rupture, the athlete had another MRI that showed a contiguous ACL that was taut with normal thickness (i.e., no elongation), and had a normal ACL appearance with hypointense signal (see Figure 1E). The Anterior Cruciate Ligament OsteoArthritis Score (ACLOAS) is an MRI classification used to assess healing of the ACL and determine long‐term changes in the whole knee joint [19]. The patient's ACL appearance on MRI indicates an ACLOAS of 0 (corresponding to a normal ligament with hypointense signal and regular thickness and continuity). The athlete was examined clinically and had no side‐to‐side difference in passive knee laxity with unchanged results in the Lachman and pivot shift tests. The athlete reported having experienced no pain or knee instability. The athlete had returned to sport and exercise, including swimming, running, gym, and Australian rules football, without restriction. The ACL had not re‐ruptured after return to competitive cutting and pivoting sport, with no episodes of giving way or new knee injuries. The athlete reported returning to complete and unrestricted sports participation at the same elite level, with no perceived decrement in performance or fear of re‐injury. The patient reported that the knee felt the same as the uninjured contralateral knee.

## 5 Discussion

This case demonstrates the potential of the CBP to restore knee stability and function for the management of complete ACL rupture in an elite athlete. Case studies that detail non‐operative management of ACL injuries in elite and professional athletes are scarce; however, there are some cases of elite and professional‐level athletes returning to sport post ACL rupture with rehabilitation alone, and one elite hockey player managed with the CBP [16, 17, 18, 20]. These reports have limited applicability to the presented case, as none were elite‐level AFL athletes managed with the CBP, and none have included MRI evidence of healing status for an elite athlete beyond nine months, a key reason for presenting this case study. Previous case reports related to non‐operative management of ACL injuries often acknowledge that the athlete is returning to sport with an ‘ACL‐deficient knee,’ and has ‘coped’ without an ACL, whereas the case report presented highlights the potential of non‐operative treatment to result in return to sport with an ACL‐intact knee, following an isolated complete rupture of the ACL [20, 21].

The previously held belief that the ACL cannot heal due to poor blood supply has been a driving factor for the strong clinical preference of surgical intervention to restore knee stability. This long‐held belief has resulted in minimal histopathologic analysis of collagen maturation and organization in ACLs that have healed non‐operatively after traumatic ACL rupture [22]. It is hypothesized that the changes in the ACL observed over time on MRI (including the progression from slight elongation to taut and the MRI signal intensity returning to ‘normal’) may reflect the synthesis, reorganization, and remodeling of collagen within the ACL. It is known that ligamentous injuries result in remodeling and reorganization of collagen for other regions of the body, but without histopathologic analysis of the ACL in this case, we are unable to determine, based on MRI alone, if optimal collagen maturation has occurred [23]. In the future, investigating collagen distribution in the ACL over time with advanced imaging modalities such as dual‐energy computed tomography (CT), colored collagen maps, optimizing MRI sequences, and exploring the potential of T2* mapping and DTI tractography, may provide some information on the process and extent of healing in the ACL when managed with the CBP [24, 25].

The sequential improvement of the ACL appearance on MRI aligned with improvement in PROMS over time and resulted in a return to play timeframe of < 12 months from the time of injury. Despite the successful outcome of this patient, the study is limited due to its single‐case nature, the inability to arthroscopically verify the diagnosis of complete ACL rupture, and the subjective nature of PROMS. Future studies should focus on larger, controlled studies that compare the outcomes of ACL‐injured patients managed with the CBP to those managed both surgically and with rehabilitation alone.

## 6 Conclusion

The case presented invites discussion for optimization of non‐operative treatment in elite athletes with ACL injuries. The athlete's return to elite cutting, pivoting, and contact sport without re‐rupture supports the potential for the CBP as a non‐operative management option for ACL injuries in elite athletes. Multiple randomized controlled trials are now underway to assess the effectiveness of the CBP compared to rehabilitation‐alone and compared to early ACL reconstruction. Additionally, several key knowledge gaps remain that require further investigation to inform clinical recommendations.

## Author Contributions

**Keiley Mead:** conceptualization, methodology, project administration, visualization, writing – original draft. **Matthew Dowsett:** writing – review and editing. **Zoe Cass:** data curation, writing – review and editing. **Stephanie Filbay:** conceptualization, methodology, supervision, writing – review and editing.

## Funding

This case report is not part of a funded research program. No external funding was received for the preparation of this manuscript. A/Prof Stephanie Filbay is supported by a National Health and Medical Research Council (NHMRC) Investigator Grant (#1194428). Keiley Mead is supported by an Australian Government Research Training Program Scholarship.

## Ethics Statement

The University of Sydney ethics committee has advised that Clinical Case Reports are exempt from HREC review as they do not meet the definition of a human research activity, instead falling under the category of a medical/educational activity. Given that written consent from the patient has been obtained, no further ethical review was required.

## Consent

Written informed consent was obtained from the patient for the publication of this case report.

## Conflicts of Interest

The authors declare no conflicts of interest.

## Data Availability

The data that support the findings of this study are available on request from the corresponding author. The data are not publicly available due to privacy or ethical restrictions.
